# Supplementary material for: Modelling Skylarks (Alauda arvensis) to Predict Impacts of Changes in Land Management and Policy: Development and Testing of an Agent-Based Model
Source: PLoS One. 2013 Jun 6;8(6):e65803. doi: 10.1371/journal.pone.0065803 (PMC3675089; doi:10.1371/journal.pone.0065803)
Supplement: Supporting Information S4 — The skylark ODdox as a zipped archive. (ZIP) [file pone.0065803.s004.zip › Skylark_ODdox/class_simple_position_map.html]

ALMaSS Skylark ODdox: SimplePositionMap Class Reference


ALMaSS Skylark ODdox
 2.0


- Main Page
- Related Pages
- Classes
- Files

- Class List
- Class Index
- Class Hierarchy
- Class Members

Public Member Functions |
Public Attributes

SimplePositionMap Class Reference

Used to map locations of individuals for density estimates - space inefficient but good for testing.
More...

`#include <positionmap.h>`

List of all members.

|  |  |
| --- | --- |
| Public Member Functions | |
| virtual void | ClearMapValue (unsigned a\_x, unsigned a\_y) |
| virtual int | GetMapDensity (unsigned a\_x, unsigned a\_y, unsigned a\_range) |
| virtual bool | GetMapPositive (unsigned a\_x, unsigned a\_y, unsigned a\_range) |
| virtual bool | GetMapValue (unsigned a\_x, unsigned a\_y) |
| virtual void | SetMapValue (unsigned a\_x, unsigned a\_y) |
|  | SimplePositionMap () |
|  | SimplePositionMap (unsigned a\_size) |
|  | SimplePositionMap (Landscape \*L) |
| virtual | ~SimplePositionMap () |

|  |  |
| --- | --- |
| Public Attributes | |
| unsigned int | m\_maxx |
| unsigned int | m\_maxy |
| bool \* | m\_TheMap |

---

## Detailed Description

Used to map locations of individuals for density estimates - space inefficient but good for testing.

---

## Constructor & Destructor Documentation

|  |  |  |  |  |
| --- | --- | --- | --- | --- |
| SimplePositionMap::SimplePositionMap | ( |  | ) |  |

{

;// Default do nothing constructor

}

|  |  |  |  |  |  |
| --- | --- | --- | --- | --- | --- |
| SimplePositionMap::SimplePositionMap | ( | unsigned | *a\_size* | ) |  |

References ClearMapValue(), m\_maxx, m\_maxy, and m\_TheMap.

{

m\_maxx=a\_size;

m\_maxy=m\_maxx;

m\_TheMap = new bool[ m\_maxx\*m\_maxy ];

for (unsigned y=0; y<m\_maxy; y++)

{

for (unsigned x=0; x<m\_maxx; x++)

{

ClearMapValue(x,y);

}

}

}

|  |  |  |  |  |  |
| --- | --- | --- | --- | --- | --- |
| SimplePositionMap::SimplePositionMap | ( | Landscape \* | *L* | ) |  |

References ClearMapValue(), m\_maxx, m\_maxy, m\_TheMap, Landscape::SupplySimAreaHeight(), and Landscape::SupplySimAreaWidth().

{

m\_maxx=L->SupplySimAreaWidth();

m\_maxy=L->SupplySimAreaHeight();

m\_TheMap = new bool[ m\_maxx\*m\_maxy ];

for (unsigned y=0; y<m\_maxy; y++)

{

for (unsigned x=0; x<m\_maxx; x++)

{

ClearMapValue(x,y);

}

}

}

|  |  |  |  |  |
| --- | --- | --- | --- | --- |
| SimplePositionMap::~SimplePositionMap | ( |  | ) |  |

virtual

References m\_TheMap.

{

delete m\_TheMap;

}

---

## Member Function Documentation

|  |  |  |  |
| --- | --- | --- | --- |
| virtual void SimplePositionMap::ClearMapValue | ( | unsigned | *a\_x*, |
|  |  | unsigned | *a\_y* |
|  | ) |  |  |

inlinevirtual

Reimplemented in ScalablePositionMap.

References m\_maxy, and m\_TheMap.

Referenced by SimplePositionMap().

{

m\_TheMap[a\_x+a\_y\*m\_maxy]=false;

}

|  |  |  |  |
| --- | --- | --- | --- |
| virtual int SimplePositionMap::GetMapDensity | ( | unsigned | *a\_x*, |
|  |  | unsigned | *a\_y*, |
|  |  | unsigned | *a\_range* |
|  | ) |  |  |

inlinevirtual

Reimplemented in ScalablePositionMap.

References m\_maxy, and m\_TheMap.

{

int dens=0;

for (unsigned x = a\_x; x<a\_x+a\_range; x++)

{

for (unsigned y = a\_y; y<a\_y+a\_range; y++)

if (m\_TheMap[x+y\*m\_maxy]) dens++;

}

return dens;

}

|  |  |  |  |
| --- | --- | --- | --- |
| virtual bool SimplePositionMap::GetMapPositive | ( | unsigned | *a\_x*, |
|  |  | unsigned | *a\_y*, |
|  |  | unsigned | *a\_range* |
|  | ) |  |  |

inlinevirtual

Reimplemented in ScalablePositionMap.

References m\_maxy, and m\_TheMap.

{

for (unsigned x = a\_x; x<a\_x+a\_range; x++)

{

for (unsigned y = a\_y; y<a\_y+a\_range; y++)

if (m\_TheMap[x+y\*m\_maxy]) return true;

}

return false;

}

|  |  |  |  |
| --- | --- | --- | --- |
| virtual bool SimplePositionMap::GetMapValue | ( | unsigned | *a\_x*, |
|  |  | unsigned | *a\_y* |
|  | ) |  |  |

inlinevirtual

Reimplemented in ScalablePositionMap.

References m\_maxy, and m\_TheMap.

{

return m\_TheMap[a\_x+a\_y\*m\_maxy];

}

|  |  |  |  |
| --- | --- | --- | --- |
| virtual void SimplePositionMap::SetMapValue | ( | unsigned | *a\_x*, |
|  |  | unsigned | *a\_y* |
|  | ) |  |  |

inlinevirtual

Reimplemented in ScalablePositionMap.

References m\_maxy, and m\_TheMap.

{

m\_TheMap[a\_x+a\_y\*m\_maxy]=true;

}

---

## Member Data Documentation

unsigned int SimplePositionMap::m\_maxx

Referenced by ScalablePositionMap::ScalablePositionMap(), and SimplePositionMap().

unsigned int SimplePositionMap::m\_maxy

Referenced by ClearMapValue(), ScalablePositionMap::ClearMapValue(), GetMapDensity(), ScalablePositionMap::GetMapDensity(), GetMapPositive(), ScalablePositionMap::GetMapPositive(), GetMapValue(), ScalablePositionMap::GetMapValue(), ScalablePositionMap::ScalablePositionMap(), SetMapValue(), ScalablePositionMap::SetMapValue(), and SimplePositionMap().

bool\* SimplePositionMap::m\_TheMap

Referenced by ClearMapValue(), ScalablePositionMap::ClearMapValue(), GetMapDensity(), ScalablePositionMap::GetMapDensity(), GetMapPositive(), ScalablePositionMap::GetMapPositive(), GetMapValue(), ScalablePositionMap::GetMapValue(), ScalablePositionMap::ScalablePositionMap(), SetMapValue(), ScalablePositionMap::SetMapValue(), SimplePositionMap(), and ~SimplePositionMap().

---

The documentation for this class was generated from the following files:

- positionmap.h
- PositionMap.cpp


- SimplePositionMap
- Generated on Thu Jan 10 2013 13:15:37 for ALMaSS Skylark ODdox by
   1.8.1.1
